# 磁性氮化碳复合材料的制备及其对磷酸化肽的富集

**DOI:** 10.3724/SP.J.1123.2023.11007

**Published:** 2024-06-08

**Authors:** Liyan JIANG, Weilu ZHANG, Lu ZHAO, Lianghai HU

**Affiliations:** 吉林大学生命科学学院, 吉林 长春 130012; College of Life Sciences, Jilin University, Changchun 130012, China

**Keywords:** 氮化碳, 磁性固相萃取, 基质辅助激光解吸电离飞行时间质谱, 磷酸化肽, 富集, carbon nitride, magnetic-solid phase extraction (MSPE), matrix-assisted laser desorption/ionization time-of-flight mass spectrometry (MALDI-TOF-MS), phosphopeptide, enrichment

## Abstract

蛋白质的磷酸化在细胞信号传导和疾病发生发展中起着重要作用,但磷酸化的动态变化和低丰度的特点使得对其直接分析有着较大的困难。为了解决磷酸化肽难离子化、检测丰度低的瓶颈问题,本研究制备了一种磁性氮化碳复合材料,结合基质辅助激光解吸电离飞行时间质谱(MALDI-TOF-MS),建立了一种对复杂样品中低丰度磷酸化肽富集与分析的方法。采用电子显微镜、红外光谱分析及X射线衍射分析等手段对合成的磁性氮化碳材料进行表征。以酪蛋白酶解产物为实验模型,发现磁性氮化碳材料能够实现对磷酸化肽的高选择性富集和高灵敏度检测,检出限为0.1 fmol。选择脱脂牛奶、人唾液和人血清为实际分析样品,发现磁性氮化碳材料对微量蛋白生物样品中磷酸化肽的分析具有较高的应用潜力。

## Abstract (en)

Protein phosphorylation plays an important role in cellular signaling and disease development. Advances in mass spectrometry-based proteomics have enabled qualitative and quantitative phosphorylation studies as well as in-depth biological explorations for biomarker discovery and signaling pathway analysis. However, the dynamic changes that occur during phosphorylation and the low abundance of target analytes render direct analysis difficult because mass spectral detection offers no selectivity, unlike immunoassays such as Western blot and enzyme-linked immunosorbent assay (ELISA). The present study aimed to solve one of the key problems in the specific and efficient isolation of phosphorylated peptides. A method based on a magnetic carbon nitride composite coupled with matrix-assisted laser desorption/ionization time-of-flight mass spectrometry (MALDI-TOF-MS) was developed for the enrichment and analysis of phosphopeptides with low abundance in complex samples. Magnetic carbon nitride composite was synthesized and characterized by electron microscopy, infrared spectroscopy, and X-ray diffractometry. The composite showed a well-distributed two-dimensional layered structure and functional groups with excellent paramagnetic performance. Two classical phosphoproteins, namely, *α*- and *β*-caseins, were selected as model phosphorylated samples to assess the performance of the proposed enrichment technique. The magnetic carbon nitride composite exhibited high selectivity and sensitivity for phosphopeptide enrichment. The limit of detection was determined by MALDI-TOF-MS analysis to be 0.1 fmol. The selectivity of the method was investigated using the digest mixtures of *α*-casein, *β*-casein, and bovine serum albumin (BSA) with different mass ratios (1∶1∶1000, 1∶1∶2000, and 1∶1∶5000). Direct analysis of the samples revealed the dominance of spectral signals from the abundant peptides in BSA. After enrichment with the magnetic carbon nitride composite, the high concentration of background proteins was washed away and only the signals of the phosphopeptides were captured. The signals from the casein proteins were clearly observed with little background noise, indicating the high selectivity of the composite material. The robustness of the method was tested by assessing the reusability of the same batch of magnetic carbon nitride materials over 20 cycles of enrichment. The composite showed nearly the same enrichment ability even after several cycles of reuse, demonstrating its potential applicability for a large number of clinical samples. Finally, the method was applied to the analysis of phosphopeptides from several commonly used phosphoprotein-containing samples, including skimmed milk digest, human serum, and human saliva; these samples are significant in the analysis of food quality, disease biomarkers, and liquid biopsies for cancer. Without enrichment, no phosphopeptide was detected because of the high abundance of nonphosphopeptide materials dominating the spectral signals obtained. After pretreatment with the developed magnetic carbon nitride composite, most of the phosphosites were identified with high selectivity and sensitivity via MALDI-TOF-MS. These results revealed the practicality of the developed approach for clinical applications. In addition, our method may potentially be employed for phosphoproteomics with real complex biological samples.

蛋白质磷酸化是重要的蛋白质翻译后修饰方式之一,协调细胞的生长、分化和凋亡等多种细胞功能,具有高度动态和不均一性的特点,同时调节细胞信号转导和蛋白质的行为^[1]^。蛋白质磷酸化异常与疾病密切相关,有些蛋白质常被用作生物标志物^[2]^,临床样本中靶向磷酸肽的特性对于研究疾病机制和评估可能的疾病生物标志物至关重要^[3⇓-5]^。生物样本中的磷酸化通常发生在亚化学计量水平,加之磷酸基倾向于失去质子携带负电荷,磷酸化肽倾向于具有较低的电离效率,以及大量非磷酸化肽的背景存在,导致磷酸化蛋白形态通常以较低丰度存在,并具有很高的动态范围和微异质性,使得蛋白质磷酸化的质谱分析有着较大的挑战^[6]^。因此,发展对磷酸化蛋白或磷酸化肽具有高效捕获能力的富集材料是磷酸化修饰定性定量分析的前提。

近年来,随着生物医学、环境科学、材料科学等学科的交叉发展,磷酸化蛋白与多肽的富集新方法研究也得到了快速发展。磁性固相萃取是一种方便快捷的前处理技术,被广泛应用于生物分离领域^[7,8]^,通常选用Fe_3_O_4_微球作为载体进行功能化修饰,在外加磁场的作用下实现生物样品与材料的快速分离,此外,还可将Fe_3_O_4_和其他结构的材料进行复合,提高其比表面积和吸附动力学,从而增强对目标分析物的捕获效率和灵敏度。目前,已有多种基于不同磷酸化肽富集机理的功能化磁性材料被合成并用于富集样品中的目标肽段^[9⇓-11]^,如Ti^4+^修饰的磁性纳米材料对磷酸化肽的富集选择性明显提高^[12]^,锆基金属有机骨架材料固定于多巴胺修饰的磁性纳米颗粒已经成功地应用于人唾液样品中内源磷酸化肽的提取^[13]^,基于TiO_2_内核的磁性分子印迹材料可对特定序列的磷酸多肽亚型进行高选择性富集^[14]^。

氮化碳(g-C_3_N_4_)具有罕见且独特的离域共轭化学结构,是典型的氮掺杂碳结构材料,具有良好的耐热性、耐化学稳定性和硬度高、对可见光响应好等优势,目前作为一种无金属材料被广泛应用于环境修复等方面,引起了人们极大的兴趣^[15]^。g-C_3_N_4_的离域*π*键可以对磷酸化肽产生特异性吸附,能够实现对目标物的高选择性富集,与未剥离g-C_3_N_4_相比,剥离的g-C_3_N_4_纳米片具有大比表面积,且呈现出明显的2D层状结构,具有良好的吸附性能和丰富的化学位点,可以显著增加材料中磷酸化肽的结合位点,是一种新型磷酸化肽富集材料的潜在载体^[16]^。

基于Fe_3_O_4_磁性纳米粒子容易合成、生物友好和超顺磁性等优势,考虑其本身对于特定目标物的分离不具备特异性,结合氮化碳通过离域*π*键的相互作用吸附不同目标分子的优良特性,本研究发展了磁性氮化碳复合材料,用于磷酸化肽的富集,结合基质辅助激光解吸电离飞行时间质谱(MALDI-TOF-MS),可对复杂生物样品中的磷酸化肽进行快速高灵敏的检测,为磷酸化蛋白异常引起的疾病标志物与机制研究提供了新的分析方法。

## 1 实验部分

### 1.1 仪器、试剂与材料

Nicolet iS5型傅里叶变换红外光谱仪(FT-IR, Thermo Nicolet公司,美国); H-7650型透射电子显微镜(TEM,日立公司,日本); Smartlab X-射线粉末衍射仪(XRD,理学公司,日本);多功能振动样品磁强计(VSM,量子科学仪器贸易公司,美国)。5800基质辅助激光解吸电离飞行时间质谱仪(SCIEX,美国)。

六水合三氯化铁(FeCl_3_·6H_2_O)、碳酸氢铵(NH_4_HCO_3_)、三聚氰胺(C_3_H_6_N_6_)、乙醇、四水合氯化亚铁(FeCl_2_·4H_2_O)、三氟乙酸(TFA)、2,5-二羟基苯甲酸(DHB)、乙腈(ACN)和氨水(质量分数28%)购自安耐吉化学试剂有限公司;*α*-酪蛋白、*β*-酪蛋白、牛血清蛋白和胰蛋白酶购自Sigma试剂有限公司。血清、唾液样品来源于吉林大学第二医院。研究经吉林大学第二医院医学伦理委员批准,批准号为SB-2023-008。

### 1.2 Fe_3_O_4_/g-C_3_N_4_的制备

称量10 g三聚氰胺,研磨后放入坩埚中,在管式炉中于550 ℃加热2 h,得到g-C_3_N_4_。将0.6 g g-C_3_N_4_产物分散至180 mL水溶液中,超声6 h后对其进行离心干燥得到剥离的g-C_3_N_4_。

将g-C_3_N_4_剥离产物分散在500 mL乙醇-水(1∶2, v/v)溶液中,加入10 mL FeCl_3_(0.65 mol/L)、10 mL FeCl_2_(0.70 mol/L)和5 mL氨水,在80 ℃下搅拌30 min。将产物置于80 ℃的烘箱中干燥12 h,得到Fe_3_O_4_/g-C_3_N_4_^[13]^。

### 1.3 样品前处理

蛋白酶解:称取1 mg *α*-酪蛋白或*β*-酪蛋白样品,溶解于1 mL 50 mmol/L NH_4_HCO_3_溶液中,于100 ℃加热变性10 min,冷却至室温,加入20 μL 1 mg/mL胰蛋白酶,于37 ℃水浴条件下酶解16 h,然后在室温下加入2 μL甲酸终止反应。酶解液置于-20 ℃冰箱中保存备用^[17]^。

脱脂牛奶:在1 mL脱脂牛奶中加入5 mL 50 mmol/L NH_4_HCO_3_溶液,在3500 g的离心力下离心20 min,取上层清液,在100 ℃金属浴中保持10 min后,冷却至室温。加入20 μL 1 mg/mL胰蛋白酶,在37 ℃水浴中酶解16~18 h,加入20 μL甲酸终止反应。用4 mL 50 mmol/L NH_4_HCO_3_缓冲溶液稀释,以备后续分析^[18]^。

血清、唾液:取1 mL唾液和血清,加入5 mL 50 mmol/L NH_4_HCO_3_缓冲溶液,在25 ℃下孵育1 h。所得产物保存在-20 ℃以备后续分析^[19,20]^。

### 1.4 磷酸化肽的富集过程

酶解后的多肽用含50%(v/v) ACN和0.1%(v/v) TFA的水溶液进行稀释。将0.5 mg Fe_3_O_4_/g-C_3_N_4_加入到100 μL该溶液中,超声处理30 min,在磁铁的作用下分离Fe_3_O_4_/g-C_3_N_4_。用100 μL含50%(v/v) ACN和0.1%(v/v) TFA的水溶液洗涤Fe_3_O_4_/g-C_3_N_4_ 3次,然后用30 μL 5%(v/v)氨水超声处理10 min,以洗脱捕获的磷酸化肽,将得到的洗脱液进行质谱分析。

### 1.5 MALDI-TOF-MS条件

将0.5 μL肽段样品与0.5 μL DHB充分混合,点样于MALDI靶板上,室温自然结晶后进行样品的检测与分析。离子源:激光电离源,反射正离子模式;激光波长255 nm,激光能量4500 J;扫描范围:*m/z* 1000~3500^[21]^。

## 2 结果与讨论

### 2.1 Fe_3_O_4_/g-C_3_N_4_的表征

本实验首先合成了g-C_3_N_4_和g-C_3_N_4_剥离产物,利用TEM对g-C_3_N_4_和g-C_3_N_4_剥离产物的形貌进行表征。结果表明未剥离的g-C_3_N_4_呈现出明显的堆叠现象(图1a)。相比之下,g-C_3_N_4_的剥离产物分散均匀,具有明显的单层片状结构(图1b)。

**图 1:**
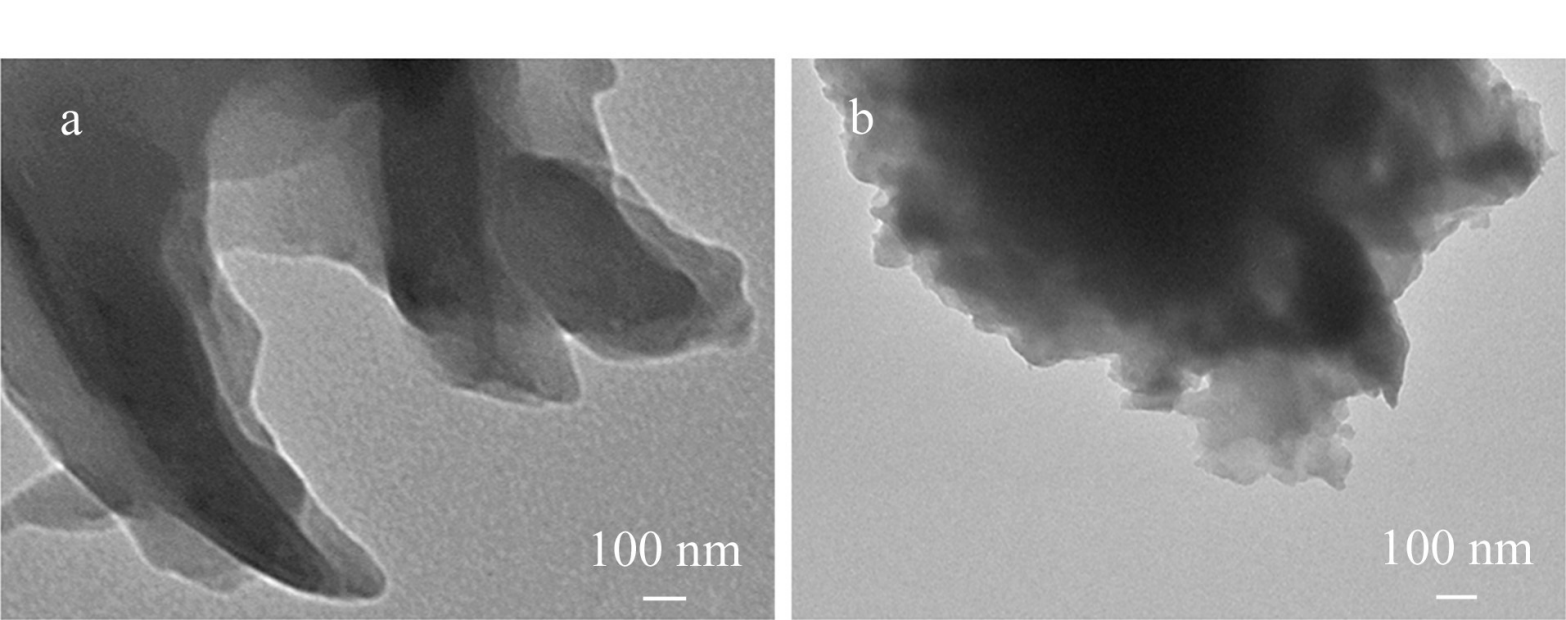
(a)g-C_3_N_4_和(b)g-C_3_N_4_剥离产物的TEM图像

利用FT-IR、XRD和VSM对Fe_3_O_4_/g-C_3_N_4_的表面基团、晶体结构及磁响应性能进行分析。观察Fe_3_O_4_/g-C_3_N_4_的FT-IR谱图,位于3385和1645 cm^-1^处的两个吸收峰为与C-N有关的固有振动,而位于586 cm^-1^处的吸收峰属于Fe-O振动(图2a)。

**图 2:**
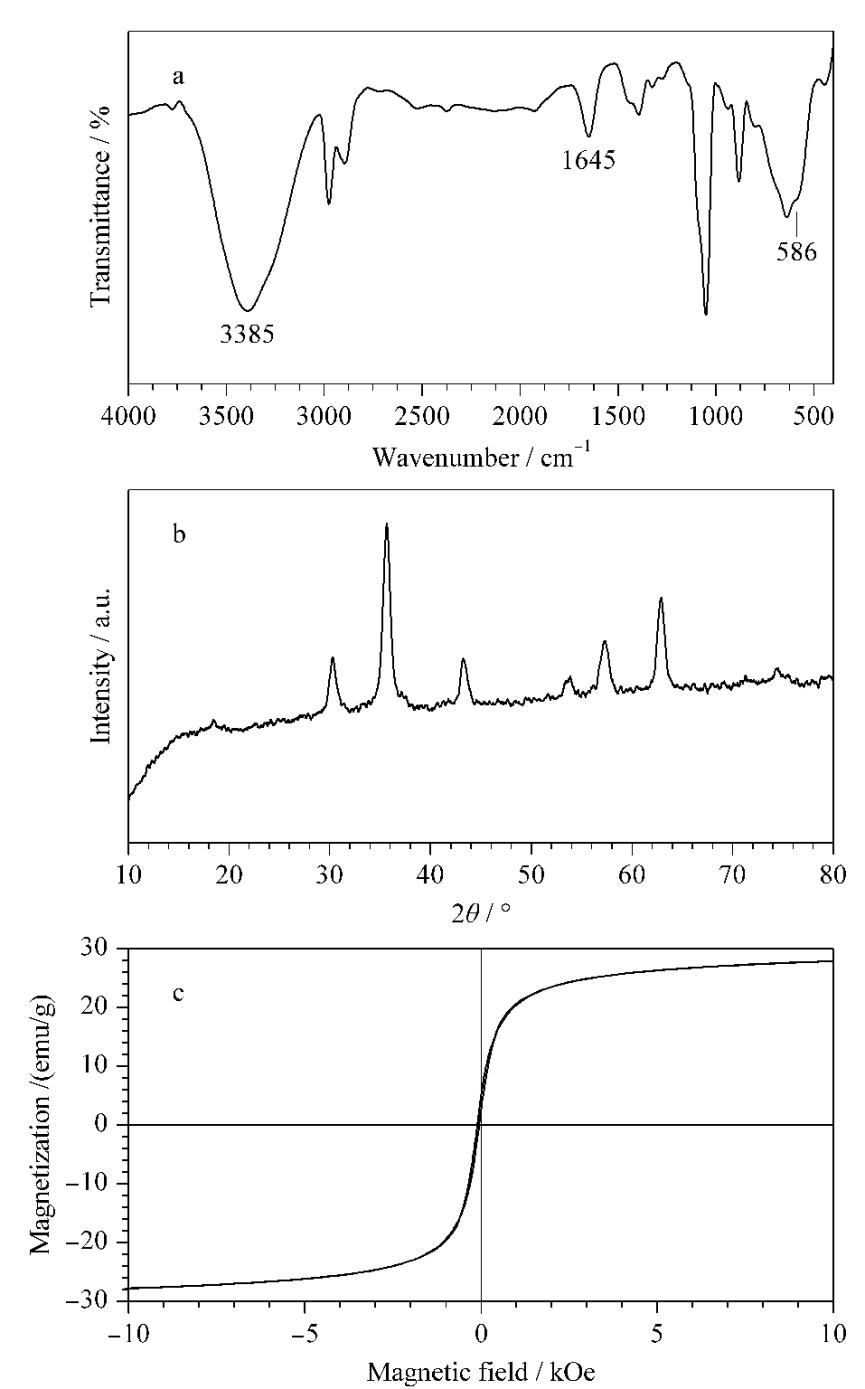
Fe_3_O_4_/g-C_3_N_4_的(a)FT-IR谱图、(b)XRD谱图 和(c)VSM谱图

Fe_3_O_4_/g-C_3_N_4_的XRD谱图也出现了明显的特征峰(图2b)。VSM谱图表明Fe_3_O_4_/g-C_3_N_4_的饱和磁化值为27.9 emu/g(图2c)。以上结果表明,Fe_3_O_4_被成功沉积在剥离的g-C_3_N_4_纳米片表面。

### 2.2 Fe_3_O_4_/g-C_3_N_4_对磷酸化肽的富集能力

实验考察了Fe_3_O_4_/g-C_3_N_4_对*α*-酪蛋白和*β*-酪蛋白1∶1混合物的酶解液中磷酸化肽的富集性能。首先考察和优化了富集和解吸条件,确定以含50%(v/v) ACN和0.1%(v/v) TFA的水溶液为缓冲溶液,5%(v/v) 氨水为解吸液。在此条件下,比较了富集前直接检测和经过Fe_3_O_4_/g-C_3_N_4_富集后检测的磷酸化肽的质谱图(图3)。通过直接分析,大量的非磷酸化肽信号出现在质谱图中(图3a),而经Fe_3_O_4_/g-C_3_N_4_富集后,非磷酸化肽的信号显著降低,可以明显观察到多条磷酸化肽的信号峰(图3b)。结果表明,制备的氮化碳磁性材料能够有效捕获酪蛋白酶解液中的磷酸化肽。

**图 3:**
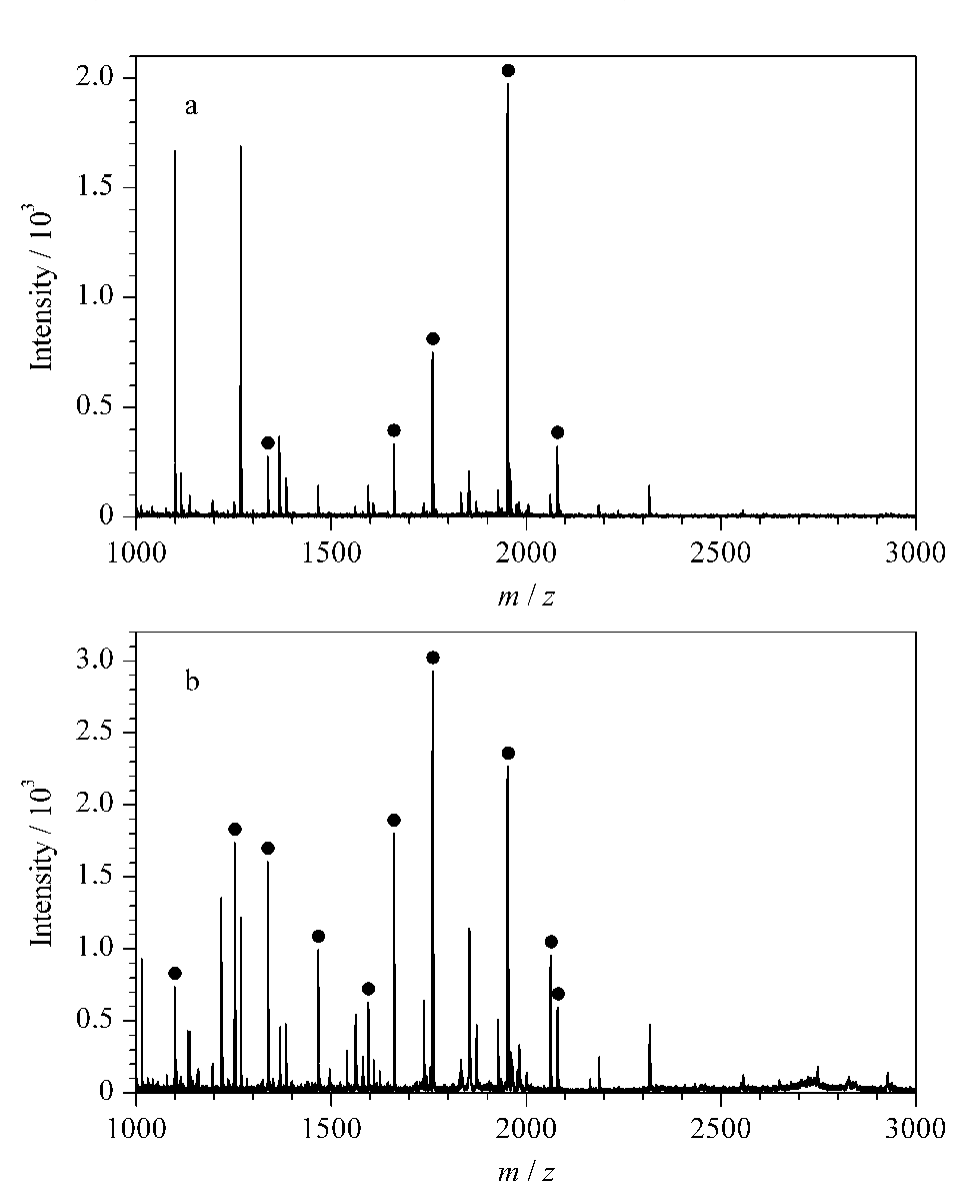
酪蛋白酶解液的MALDL-TOF-MS谱图

由于复杂生物样品中磷酸化肽的浓度较低,因此要求检测方法具有较高的检测灵敏度。为了评价基于Fe_3_O_4_/g-C_3_N_4_富集磷酸化肽的MALDI-TOF-MS方法的灵敏度,分别选取10、1和0.1 fmol酪蛋白的胰蛋白酶解液作为检测样品(图4左)。结果表明,当酪蛋白量为0.1 fmol时,仍可以观察到具有较强信号的磷酸化肽,检出限低至0.1 fmol,证明本实验方法具有较高的检测灵敏度。实验还考察了Fe_3_O_4_/g-C_3_N_4_的使用重复性,以酪蛋白的胰蛋白酶解液作为检测样品,重复使用Fe_3_O_4_/g-C_3_N_4_ 20次,富集后得到的磷酸化肽信号强度与第1次使用时差别不大(图4右),表明该磁性材料可以多次重复使用,适用于大量临床样品的富集分析。

**图 4:**
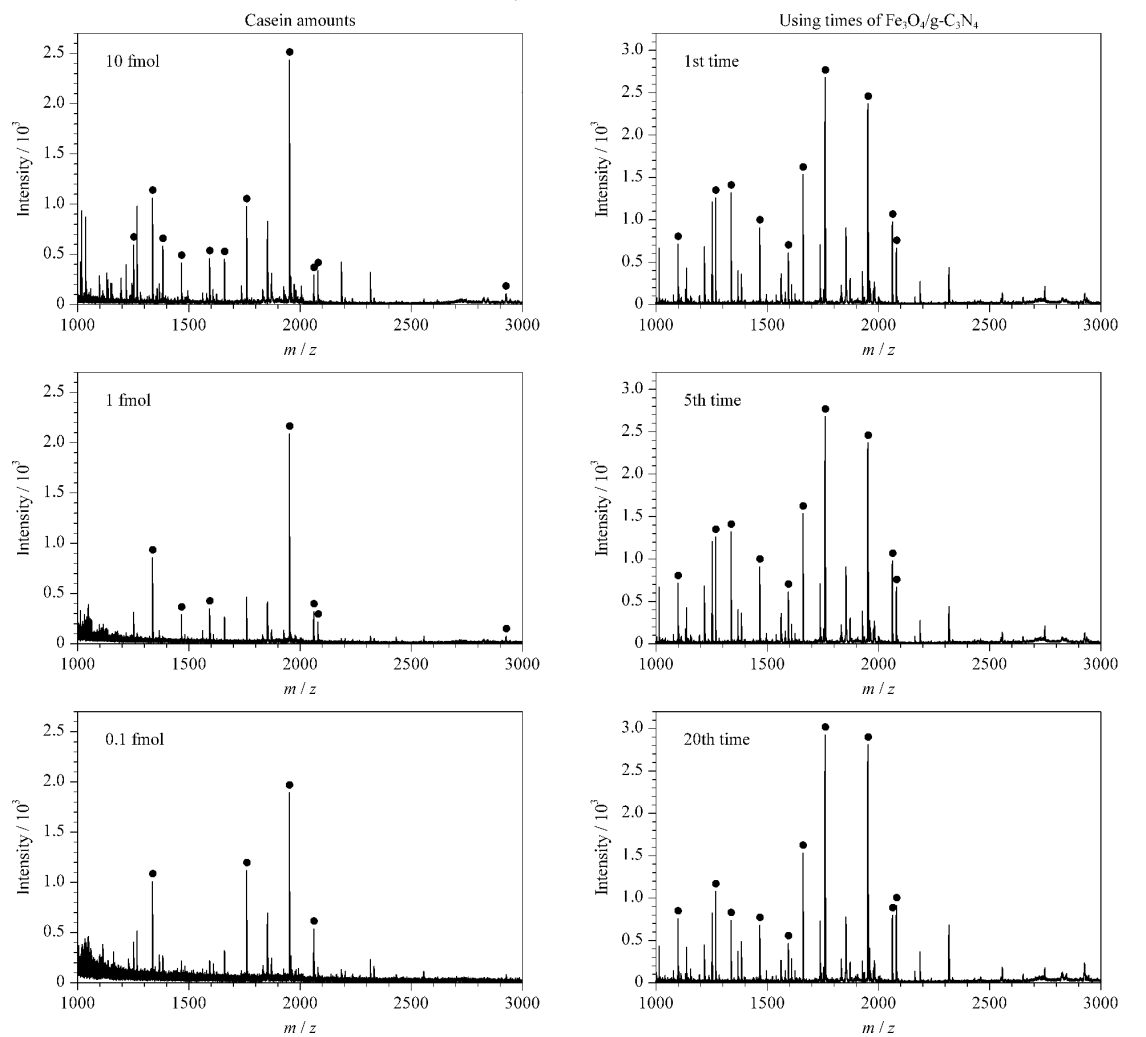
酪蛋白酶解液经Fe_3_O_4_/g-C_3_N_4_富集后的MALDI-TOF-MS谱图

为了考察Fe_3_O_4_/g-C_3_N_4_对磷酸化肽的富集选择性,将磷酸化蛋白*α*-和*β*-酪蛋白与典型的非磷酸化蛋白BSA按质量比1∶1∶1000 (图5a、5b)、1∶1∶2000 (图5c、5d)、1∶1∶5000 (图6e、6f)混合作为复杂样品,其酶解液的质谱分析结果表明,在直接分析所得质谱图中未观察到磷酸化肽的信号峰(图5a、5c、5e);而在经Fe_3_O_4_/g-C_3_N_4_富集后的质谱图(图5b、5d、5f)中,非磷酸肽的信号被显著抑制,磷酸化肽的信号峰非常明显,说明Fe_3_O_4_/g-C_3_N_4_在复杂样品中对磷酸化肽也具有较高的选择性。

**图 5:**
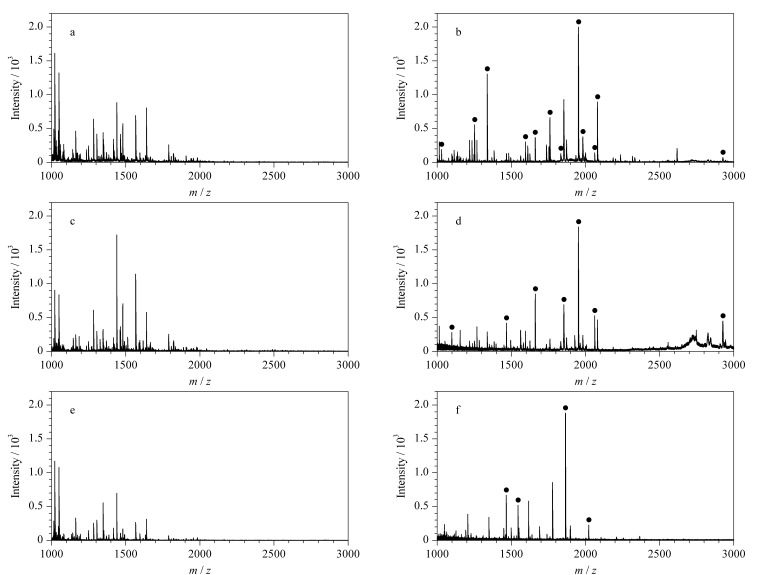
*α*-酪蛋白、 *β*-酪蛋白和牛血清蛋白混合物酶解液的MALDI-TOF-MS谱图

**图 6:**
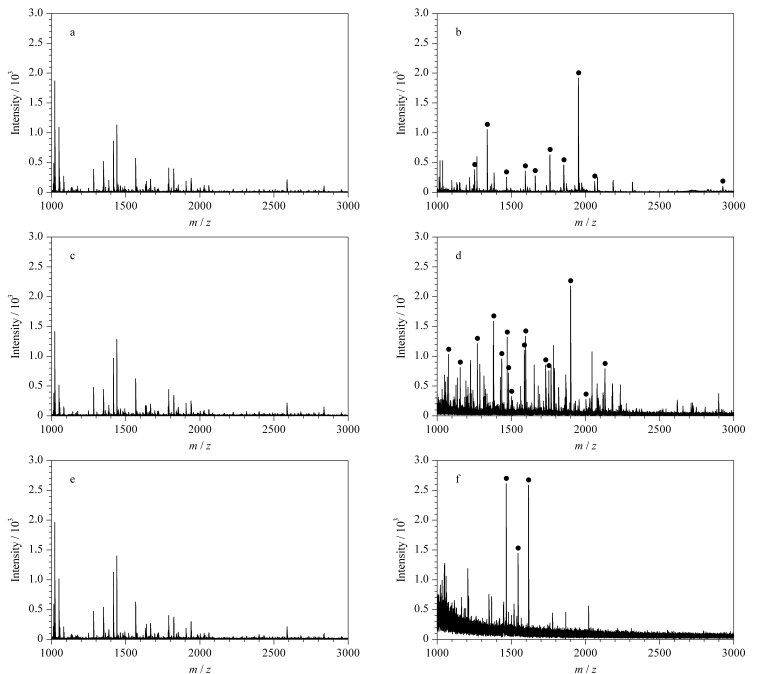
(a, b)脱脂牛奶、(c, d)人唾液和(e, f)人血清酶解物的MALDL-TOF-MS谱图

### 2.3 Fe_3_O_4_/g-C_3_N_4_对实际样品的分析

为了更好地验证该材料对复杂生物样品的适用性,选取脱脂牛奶、人血清和人唾液作为实际样品,直接对其酶解产物进行分析时,没有观察到磷酸化肽的质谱峰(图6a、6c、6e),而经Fe_3_O_4_/g-C_3_N_4_富集后,质谱图中出现了明显的磷酸化肽信号峰(图6b、6d、6f),证明该磁性材料在实际样品中具有较高的磷酸化肽富集性能,富集到的磷酸化肽详细信息见表1。

**表 1:** Fe_3_O_4_/g-C_3_N_4_富集到的脱脂牛奶、人血清和人唾液磷酸化肽的详细信息

Sample	Observed (m/z)	Number of phosphorylation site	Peptide sequence	Sample	Observed (m/z)	Number of phosphorylation site	Peptide sequence
Skimm-	1252.4	1	TVD[Mo]E[pS]TEVF	Human	1078.1	1	[pS]SEEKFLR
ed milk	1337.0	1	HIQKEDV[pS]ER	saliva	1154.3	2	[pS][pS]EEKFLR
	1465.8	1	TVDME[pS]TEVFTK		1268.6	2	D[pS][pS]EEKFLR
	1594.5	1	TVDME[pS]TEVFTKK		1380.5	1	DVPLVISDGGD[pS]E
	1660.1	1	TVDME[pS]TEVFTK		1433.9	1	[pS]HEKRHHGYR
	1760.2	0	HQGLPQEVLNENLLR		1471.5	1	DVnSS[pS]PVMLAFK
	1852.5	2	YLGEYLIVPN[pS]AEER		1481.6	1	GAPG[pS]VGPAGPRGPAGP
	1951.7	1	YKVPQLEIVPN[pS]AEER		1503.1	1	AL[pS]DTTEELTVIK
	2060.3	1	FQ[pS]EEQQQTEDELQDK		1586.2	1	GGD[pS]EQFIDEERQ
	2928.5	4	ELEELNVPGEIVESL[pS]SSEESITR		1595.6	2	D[pS][pS]EEKFLRRIG
Human	1465.3	1	D[pS]GEGDFLAEGGGVR		1731.1	1	GPPAQGG[pS]KSQSARAPPG
serum	1545.4	1	D[pS]GEGDFLAEGGGVR		1750.0	1	QPPQ[pS]StMGymGSQ
	1616.5	1	AD[pS]GEGDFLAEGGGVR		1900.1	1	GPPGSRG[pS]PGAPGPPGPPGSH
					2005.3	1	VISDGGD[pS]EQFIDEERQ
					2182.4	1	VPLVISDGGD[pS]EQFIDEER

进一步将所制备的氮化碳磁性材料与文献报道的磁性材料进行比较,见表2。从检出限及选择性数据可以看出,本工作制备的氮化碳磁性材料对复杂样品中磷酸化肽的检测灵敏度更高、选择性更好,在生物样品中痕量低丰度修饰蛋白的分析中具有较高的应用潜力。

**表 2:** 基于不同材料富集磷酸化肽的检出限比较

Enrichment material	Limit of detection/fmol	Selectivity (mass ratio)	Ref.
PP-x-Arg	10.0	β-casein/BSA (1∶100)	[22]
GF-TiO_2_-GO	10.0	β-casein/BSA (1∶100)	[23]
SPMA nanosphere	10.0	β-casein/BSA (1∶1000)	[24]
Poly(guanidinium ionic liquid)	4.0	β-casein/BSA (1∶1000)	[25]
Fe_3_O_4_/PDA/PAMA-Arg	0.2	β-casein/BSA (1∶500)	[26]
Mag-Arg	0.1	β-casein/OAV/BSA (1∶2000∶2000)	[27]
Fe_3_O_4_/g-C_3_N_4_	0.1	α-casein/β-casein/BSA (1∶1∶5000)	this work

OAV: ovalbumin.

## 3 结论

本研究开发了一种制备简单、稳定性高及生物相容性好的Fe_3_O_4_/g-C_3_N_4_磁性复合材料,并将其应用于蛋白质翻译后修饰的磷酸化肽研究。所制备的g-C_3_N_4_剥离产物分散均匀,具有明显的二维层状结构,且Fe_3_O_4_被成功沉积在剥离的g-C_3_N_4_纳米片表面,该磁性材料具有快速磁响应、亲水性和正电性。将Fe_3_O_4_/g-C_3_N_4_磁性复合材料成功应用于脱脂牛奶、人唾液及人血清3种复杂样品中磷酸化肽的检测分析,均获得了较明显的磷酸化肽样品峰,且检出限低至0.1 fmol。Fe_3_O_4_/g-C_3_N_4_磁性复合材料具有高比表面积,而且对磷酸化肽的选择性提高和负载能力增大,可以将其作为生物功能吸附剂用于选择性富集实际生物样品中的痕量磷酸化肽。该磁性复合材料具有较高的选择性和灵敏度,并可进行多次重复使用,在大量临床样品中规模化磷酸化蛋白质组学分析中具有较高的临床潜力。
